# Management of diabetes mellitus in people living with HIV: A single-center experience

**DOI:** 10.3389/fphar.2022.1082992

**Published:** 2023-01-11

**Authors:** Dario Cattaneo, Antonio Gidaro, Antonio Rossi, Andrea Merlo, Tiziana Formenti, Paola Meraviglia, Spinello Antinori, Cristina Gervasoni

**Affiliations:** ^1^ Gestione Ambulatoriale Politerapie (GAP) Outpatient Clinic, ASST Fatebenefratelli Sacco University Hospital, Milan, Italy; ^2^ Unit of Clinical Pharmacology, ASST Fatebenefratelli Sacco University Hospital, Milan, Italy; ^3^ Department of Biomedical and Clinical Sciences Luigi Sacco, University of Milan, Luigi Sacco Hospital Milan, Milan, Italy; ^4^ Division of Endocrinology, ASST Fatebenefratelli Sacco University Hospital, Milan, Italy; ^5^ Department of Infectious Diseases, ASST Fatebenefratelli Sacco University Hospital, Milan, Italy

**Keywords:** HIV, diabetes, drug-drug interactions, hypoglycemic agents, antiretroviral therapies

## Abstract

**Background:** Diabetes mellitus (DM) is more common in people living with HIV (PLWH) than in HIV-negative patients. Here we aimed to describe the response of PLWH with DM to glucose-lowering therapies in a reference hospital of northern Italy.

**Setting:** 200 PLWH and DM were identified from the database of our clinic.

**Methods:** Good control of DM was defined as having fasting glucose <130 mg/dl or HbA1c < 53 mmol/mol. The distribution of glucose-lowering therapies in PLWH was compared with that of HIV-negative patients with DM.

**Results:** Mean total fasting glucose and HbA1C were 143 ± 50 mg/dl (51% exceeding the 130 mg/dl cutoff) and 51 ± 16 mmol/mol (30% exceeding the 53 mmol/mol cutoff), respectively. PLWH were less treated with dipeptidyl peptidase-4 inhibitors (1.7% *versus* 9.6%, *p* < 0.01) and sulfonylureas (3.3% *versus* 13.2%, *p* < 0.01), being conversely more frequently treated with metformin (53.8% *versus* 37.7%, *p* < 0.01), glifozins plus metformin (7.1% *versus* 2.0%, *p* < 0.05) or insulin plus other glucose-lowering agents (5.5% *versus* 0.5%, *p* < 0.01).

**Conclusion:** An underuse of dipeptidyl peptidase-4 inhibitors was found which was, however, counterbalanced by a higher use of combination of drugs (including glifozins). A rational assessment of drug-drug interactions would contribute to a better selection of the best glucose lowering agent for each antiretroviral therapy.

## Introduction

Previous studies have shown that diabetes mellitus (DM) is more common in people living with HIV (PLWH) than in HIV-negative patients, posing the formers at high risk of micro- and macrovascular complications (Sarkar and Brown, 2021; da Cunha et al., 2020; Kousignian et al., 2021). No specific guidelines have been releases to date for the management of DM in PLWH; the metabolic targets and treatments in these patients are, therefore, similar to those in the general population (IDF Clinical Practice Recommendations for, 2017). As recently reviewed by Sarkar et al., based on studies that were not conducted in populations consisting solely of PLWH, metformin can be still considered as first-line therapy; however, for those patients with atherosclerosis, cardiovascular diseases and chronic kidney diseases, glucagon-like peptide 1 (GLP-1) receptor antagonists and sodium-glucose cotransporter-2 (SGLT-2 inhibitors) should be considered for use (Sarkar and Brown, 2021). Recently, Kousignian et al. (2021), in a monocentric cohort study involving 1496 PLWH, of which 156 with DM, reported that diagnosis of DM was missed in 38% of cases, and 47% of those patients did not reach optimal glycemic control. However, no specific information on the use of glucose-lowering therapies in this clinical setting were reported.

The aim of this current observational study is to describe the response of PLWH with DM to glucose-lowering therapies in a reference hospital of northern Italy for the treatment of HIV infection.

## Methods

The database of our clinic, with nearly 2700 PLWH on active follow-up, was retrospectively investigated to search for PLWH with a diagnosis of type 2 DM up to June 2022 (no sample size estimation was performed). Data on demographic characteristics, hematochemical analyses and medications at the last available follow-up were collected. Good control of DM was defined as having fasting glucose <130 mg/dl or HbA1c < 53 mmol/mol (IDF Clinical Practice Recommendations for, 2017; Billings et al., 2021). Hypercholesterolemia was defined as being on a lipid-lowering medication and having low-density lipoproteins (LDL) > 100 mg/dl. The distribution of glucose-lowering therapies in PLWH was compared with that of HIV-negative patients with DM, using primarily data from the annual report of national registry on the consumption of drugs in Italy, eventually confirmed by data from the Annals of the Associazione Medici Diabetologi (AMD). (Agenzia Italiana del Farmaco, 2019; Annali Associazione Medici Diabetologi 2020, 2020).

This study was conducted using data collected for clinical purposes, all of which had been previously made anonymous in accordance with the requirements of the Italian Personal Data Protection Code (Legislative Decree No. 196/2003) and the general authorizations issued by the Italian Data Protection Authority. Written informed consent for medical procedures/interventions performed for routine treatment purposes was collected for each patient.

The frequency distribution data are expressed as absolute numbers and percentages, while all other measures are expressed as mean values ± standard deviations. Differences in the distribution of glucose-lowering therapies in PLWH and in HIV-negative patients were tested using the chi-square statistic with Yates correction. *p* values less than 0.05 were considered to denote statistical significance.

## Results

200 PLWH and type 2 DM were identified from the database of our clinic and were included in the analysis. They were mostly men (82.5%), with mean age of 64 ± 9 years) (Table 1). The majority were treated with triple antiretroviral regimens (66%), based on tenofovir (43%) and HIV integrase inhibitors (70%). The patients had good immune-virologic control (95% had HIV-RNA <20 copies/ml; CD4^+^ 720 ± 361 cells/microL), preserved renal (serum creatinine 1.2 ± 1.7 mg/L) and liver (AST 37 ± 35 IU/ml, GGT 48 ± 46 IU/ml) functions.

**TABLE 1:** Demographic and hematochemical characteristics of the 200 people living with HIV and diabetes mellitus.

Characteristics	Data
Total patients	200
Age, years	64 ± 9
Female patients, n (%)	35 (17.5%)
Patients with VL > 50 copies/mL, n (%)	11 (5.5%)
VL, copies/mL (*n* = 11)	166 ± 194
CD4 count, cells/mm^3^	720 ± 361
Serum creatinine, mg/dL	1.2 ± 1.7
ALT, IU/mL	37 ± 35
GGT, IU/mL	48 ± 46
Serum glucose (visit -1), mg/dL	148 ± 56
HbA1c (visit -1), mmol/mol	51 ± 17
Serum glucose (last visit), mg/dL	143 ± 50
Patients with glucose >130 mg/dl, n (%)	101 (50.5%)
HbA1c (last visit), mmol/mol	51 ± 16
Patients with HbA1c > 53 mmol/mol, n (%)	59 (29.5%)
Total cholesterol, mg/dL	173 ± 44
Patients with cholesterol >200 mg/dl, n (%)	44 (22%)
Triglycerides, mg/dL	178 ± 135
HDL-cholesterol, mg/dL	44 ± 12
LDL-cholesterol, mg/dL	95 ± 38
Patients with LDL>130 mg/dl, n (%)	25 (12.5%)
VL, viral load; ALT, alanine aminotransferase; GGT, gamma glutamyl transferase; HbA1c, glycosylated hemoglobin; HDL, high-density lipoproteins; LDL, low-density lipoproteins	

Mean total fasting glucose and HbA1C were 143 ± 50 mg/dl (51% exceeding the 130 mg/dl cutoff) and 51 ± 16 mmol/mol (30% exceeding the 53 mmol/mol cutoff), respectively. Mean triglycerides, LDL and HDL were 178 ± 135, 95 ± 38 and 44 ± 12 mg/dl, respectively. Sixty-three percent had LDL <100 mg/dl (25% had LDL <70 mg/dl).

Eighteen (9%) out of the 200 PLWH with a confirmed diagnosis of DM were treated only with diet and physical activity (*versus* 5.4% in HIV-negative DM patients); the remaining were mainly treated with metformin (53.8%) or insulin (20.9%) monotherapies.

Some differences were found in the use of glucose-lowering drugs between PLWH and DM and HIV-negative patients (Table 2). In particular, PLWH are less treated with dipeptidyl peptidase-4 (DPP4) inhibitors (1.7% *versus* 9.6%, *p* < 0.01) and sulfonylureas (3.3% *versus* 13.2%, *p* < 0.01), being conversely more frequently treated with metformin (53.8% *versus* 37.7%, *p* < 0.01), glifozins plus metformin (7.1% *versus* 2.0%, *p* < 0.05) or insulin plus other glucose-lowering agents (5.5% *versus* 0.5%, *p* < 0.01). These trends were roughly confirmed also when considering national data from a different source (sulfonylureas 16.2%, DPP4 inhibitors 21.1%, glifozins 9.5%, GLP-1 receptor agonists 5.8%, glitazones 4.3%, glinides 3.6%) (Annali Associazione Medici Diabetologi 2020, 2020).

**TABLE 2:** Therapies for the treatment of HIV, dyslipidemias and diabetes mellitus.

Characteristics	PLWH and DM	HIV-negative patients with DM [9]
Total patients	200	—
Dual antiretroviral therapies, n (%)	68 (34%)	—
Triple antiretroviral therapies, n (%)	132 (66%)	—
Tenofovir-based antiretroviral therapies, n	86	—
Abacavir-based antiretroviral therapies, n	34	—
INI-based antiretroviral therapies, n	139	—
NNRTI-based antiretroviral therapies, n	58	—
Boosted-based antiretroviral therapies, n	53	—
Patients on lipid-lowering therapies, n (%)	128 (64%)	—
- statin monotherapy, n (%)	68 (53%)	—
- dietary supplements, n (%)	27 (21%)	—
- statin plus fibrate, n (%)	9 (7%)	—
- fibrate monotherapy, n (%)	9 (7%)	—
- statin plus omega-3 PUFA, n (%)	4 (3%)	—
- omega-3 PUFA monotherapy, n (%)	3 (2%)	—
- ezetimibe monotherapy, n (%)	3 (2%)	—
- statin plus ezetimibe, n (%)	3 (2%)	—
- fibrate plus omega-3 PUFA, n (%)	2 (2%)	—
Patients on glucose-lowering therapies, n (%)	182 (91%)	100%
- Metformin, %	53.8%**	37.7%
- Insulins, %	20.9%	23.6%
- Glifozins plus metformin, %	7.1%*	2.0%
- Insulin plus other glucose-lowering drugs, %	5.5%**	0.5%
- GLP-1 analogs, %	4.4%	3.9%
- Sulfonylureas, %	3.3%**	13.2%
- Glinides, %	2.7%	3.5%
- DPP4 inhibitors, %	1.7%**	9.6%
- Glitazones, %	0.6%	2.7%
- Glifozins monotherapy, %	0%	2.0%
- Other	0%	1.3%
DM, diabetes mellitus; INI, integrase inhibitor; NNRTI, non-nucleoside reverse transcriptase inhibitor; PUFA, polyunsaturated fatty acids; GLP-1, glucagon-like peptide 1; DPP4, dipeptidyl peptidase 4; **p < 0.01 and *p < 0.05 *versus* HIV-negative patients		

## Discussion

A good glycemic control was observed in our cohort of PLWH, with a large part of patients (70%) reaching the HbA1c targets established by international guidelines. This observation is in line with the one from Kousignian et al. (2021), which reported that nearly 60% of PLWH treated for DM reached the HbA1c targets. Conversely, only 25% of PLWH from our hospital *versus* 94% from the French cohort reached the target of LDL cholesterol <70 mg/dl. This is an unexpected and somewhat disappointing finding considering that 64% of our patients *versus* 38% of the French patients were on lipid-lowering therapies. At this stage we can only speculate that these results might have been related to differences in the use of more/less aggressive pharmacological treatments and/or by biases in the patients’ selection. As matter of fact, our data are totally superimposable with those from the 2020 Annals of AMD, reporting that 25.9% of patients with DM had LDL <70 mg/dl (Annali Associazione Medici Diabetologi 2020, 2020).

Some differences were found on the use of glucose-lowering drugs, with a significant trend for less use of sulfonylureas and DPP4 inhibitors in PLWH compared with the national database of HIV-negative patients with DM. It can be hypothesized that this could have been driven by the risk of potential clinically relevant drug-drug interactions (DDIs) between sulfonylureas and antiretroviral regimens according to the HIV drug interaction checker from the University of Liverpool (available at https://www.hiv-druginteractions.org/). Potential DDIs can be predicted also between saxagliptin and booster-based regimens (ritonavir or cobicistat) or between all DPP-4 inhibitors and the older non-nucleoside reverse transcriptase inhibitors (efavirenz, etravirine, nevirapine). However, bearing in mind that efavirenz, etravirine and nevirapine, as well as boosted protease inhibitor, are now considered as second choice antiretroviral treatments, as an alternative theory it can be hypothesized that the differences in the use of DPP4 inhibitors between PLWH and HIV-negative patients might be driven by an unjustified fear of DDIs, as previously demonstrated for antipsychotics, antidepressant, direct-acting anticoagulants or statins (Cattaneo et al., 2020a; Cattaneo et al., 2020b; Courlet et al., 2020). Interestingly, PLWH were treated more with combinations of drugs, including also the latest generation ones such as glifozins. This could be explained by an increased need of disease control in this high cardiovascular risk population, or more stringent controls in PLWH compared to the general population.

This is a retrospective analysis of data collected per clinical practice in all PLWH with a diagnosis of type 2 DM in our clinic. Therefore, a potential selection bias cannot be excluded. In conclusion, an underuse of DPP4 inhibitors was found in HIV-infected patients from our clinic which was, however, counterbalanced by a higher use of combination of drugs (including glifozins). A rational assessment of DDIs would contribute to a better selection of the best glucose lowering agent for each antiretroviral therapy.

## Data Availability

The data that support the findings of this study are available on reasonable request from the corresponding author.

## References

[B7] Agenzia Italiana del Farmaco (2019). L’uso dei farmaci in Italia. Rapporto nazionale 2019. https://www.aifa.gov.it/documents/20142/1205984/rapporto-osmed-2019.pdf (Accessed December 30, 2021).

[B1] Annali associazione medici diabetologi 2020. https://posta.hsacco.it/service/home/∼/?auth=co&loc=it&id=109775&part=2 , December 30, 2021, 2021.

[B2] Billings L. K. Agner B. F. R. Altuntas Y. Gron R. Halladin N. Klonoff D. C., (2021). The benefit of insulin degludec/liraglutide (ideglira) compared with basal-bolus insulin therapy is consistent across participant subgroups with type 2 diabetes in the DUAL VII randomized trial. J. Diabetes Sci. Technol 15, 636–645. 10.1177/1932296820906888 32107930 PMC8120051

[B3] Cattaneo D. Baldelli S. Resnati C. Giacomelli A. Meraviglia P. Minisci D., (2020). Evaluation of the concentrations of psychotropic drugs in HIV-infected versus HIV-negative patients: Potential implications for clinical practice. World J. Biol. Psychiatry 21, 651–657. 10.1080/15622975.2018.1500032 30058430

[B4] Cattaneo D. Formenti T. Gidaro A. Merlo A. Gervasoni C. (2020). Use of direct oral anticoagulants in people living with HIV: A single-center experience. Semin. Thromb. Hemost 46, 999–1001. 10.1055/s-0040-1718398 33368115

[B5] Courlet P. Livio F. Alves Saldanha S. Scherrer A. Battegay M. Cavassini M., (2020). Real-life management of drug-drug interactions between antiretrovirals and statins. J. Antimicrob. Chemother 75, 1972–1980. 10.1093/jac/dkaa099 32240298

[B6] da Cunha G. H. Franco K. B. Galvão M. T. G. Lima M. A. C. Fontenele M. S. M. Siqueira L. R., (2020). Diabetes mellitus in people living with HIV/AIDS: Prevalence and associated risk factors. AIDS Care 32, 600–607. 10.1080/09540121.2019.1695727 31760760

[B8] IDF clinical practice recommendations for managing type 2 diabetes in primary care international diabetes federation 2021. file:///C:/Users/utente/Downloads/IDF-T2D-CPR-2017-print.pdf , December 30, 2021 . 10.1016/j.diabres.2017.09.002 28962686

[B9] Kousignian I. Sautereau A. Vigouroux C. Cros A. Kretz S. Viard J. P., (2021). Diagnosis, risk factors and management of diabetes mellitus in HIV-infected persons in France: A real-life setting study. PLoS One 16, e0250676. 10.1371/journal.pone.0250676 33990121 PMC8121550

[B10] Sarkar S. Brown T. T. (2021). Diabetes in people with HIV. Curr. Diab Rep 21, 13. 10.1007/s11892-021-01382-8 33730223 PMC8208107

